# Effects of ginsenosides on bone remodelling for novel drug applications: a review

**DOI:** 10.1186/s13020-020-00323-z

**Published:** 2020-05-06

**Authors:** Nan Yang, Dingkun Liu, Xiao Zhang, Jianing Li, Mi Wang, Tongtong Xu, Zhihui Liu

**Affiliations:** Department of Prosthodontics, Stomatology Hospital of Jilin University, Changchun, 130021 Jilin China

**Keywords:** Panax, Ginsenosides, Osteogenesis, Osteoblasts, Osteoclasts, Bone resorption, Bone remodelling

## Abstract

**Background:**

Ginsenosides are pharmacologically active compounds that are often extracted from the Panax plant for their medicinal properties. Ginsenosides have multiple effects, including antitumor effects which have been widely studied. In recent years, studies have found that ginsenosides promote proliferation and osteogenesis of osteoblast-related cells, as well as inhibit the activity of osteoclasts.

**Main body:**

We briefly introduces the molecules and BMP, WNT, and RANKL signalling pathways involved in bone formation and bone resorption. Next, recent studies on the mechanism of action of ginsenosides in bone remodelling are reviewed from three perspectives: the effects on proliferation of osteoblast-related cells, effects on osteogenesis and effects on osteoclasts. To expedite the development of drugs containing ginsenosides, we summarize the multiple beneficial roles of various types of ginsenosides in bone remodelling; including the promotion of bone formation, inhibition of bone resorption, and anti-inflammatory and antioxidant effects.

**Conclusion:**

Many ginsenosides can promote bone formation and inhibit bone resorption, such as Rb1, Rb2 and Re. Ginsenosides have the potential to be new drugs for the treatment of osteoporosis, promote fracture healing and are strong candidates for cytokines in the tissue-engineered bone. This review provides a theoretical basis for clinical drug applications and proposes several future directions for exploring the beneficial role of ginseng compounds in bone remodelling.

## Background

**Fig. 1:**
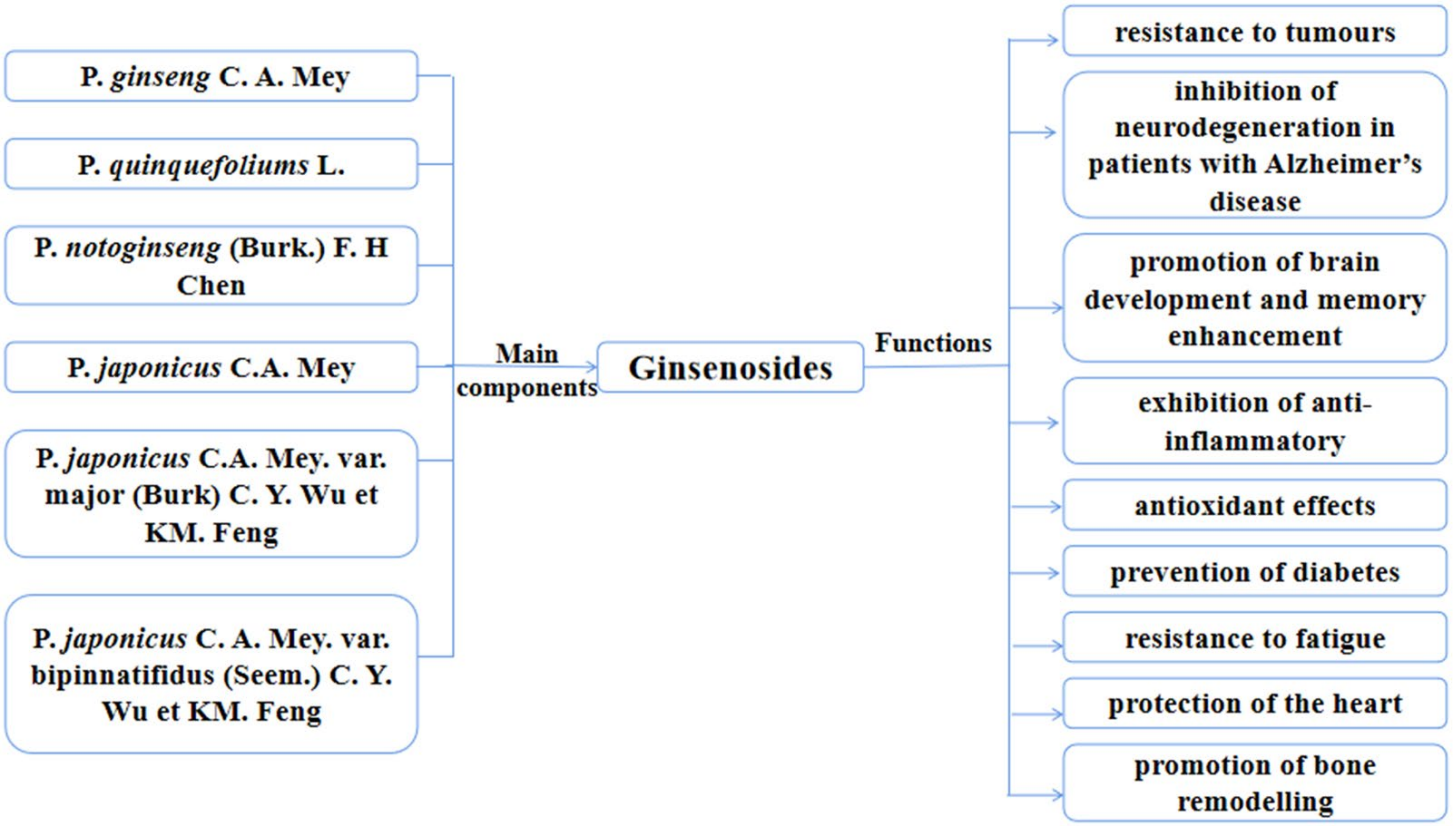
Ginsenosides are the main components of the six Panax plants and have multiple functions

Ginsenosides are the main pharmacologically active compounds present in plants of the genus Panax (ginseng), which belongs to the Araliaceae family. The main medicinal plants of Panax include *P. ginseng* C. A. Mey, *P. quinquefolius* L, *P. notoginseng* (Burk.) F. H Chen, *P. japonicus* C.A. Mey, *P. japonicus* C.A. Mey. var. major (Burk) C. Y. Wu et KM. Feng and *P. japonicus* C. A. Mey. var. bipinnatifidus (Seem.) C. Y. Wu et KM. Feng; among these, *P. ginseng* C. A. Mey., *P. quinquefolium* L., and *P. notoginseng* (Burk.) F. H. Chen. are most widely used. Ginsenosides are mostly concentrated in the roots, leaves, and flower buds of ginsengs. Petkov [1] first reported the pharmacological properties of *P. ginseng* extracts in the 1950s and since then, over 6000 articles have been published on the traditional uses, chemical constituents, and biological and pharmacological effects of ginseng. Ginsenosides are almost non-toxic to normal human cells, while their natural properties (Fig. 1) include resistance to tumours [2], inhibition of neurodegeneration in patients with Alzheimer’s disease [3], promotion of brain development and memory enhancement [4], exhibition of anti-inflammatory [5] and antioxidant effects [6], prevention of diabetes [7], resistance to fatigue [8], and protection of the heart [9], etc. In recent years, studies have found that ginsenosides also promote cell proliferation and osteogenesis, as well as inhibit osteoclasts.

The review explores the molecular mechanisms of ginsenosides that affect bone remodelling, and provide a theoretical basis for novel applications of ginsenosides as drugs. Based on the type and molecular structure of ginsenosides, this review discusses the effects and mechanisms of ginsenosides while considering their ability to promote cell proliferation and osteogenesis, as well as inhibit osteoclast formation.

## Main types of ginsenosides that affect bone remodelling

**Fig. 2:**
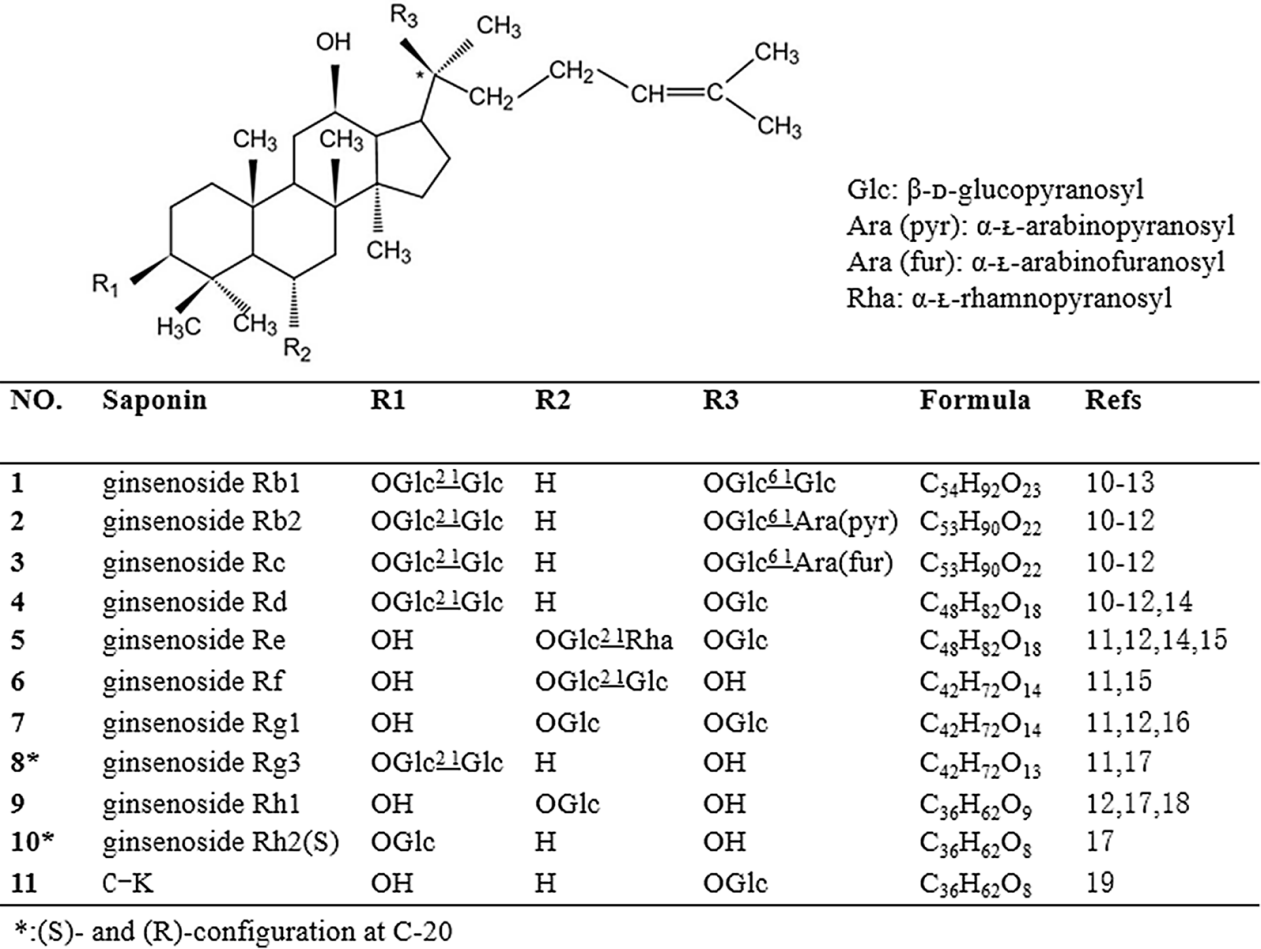
The structural formula of ginsenosides with functions related to bone remodelling

Ginsenosides are mainly extracted from *P. ginseng* C. A. Mey, *P. quinquefolium* L, and *P. notoginseng* (Burk.) F. H Chen. Based on their diverse structural characteristics, ginsenosides can be divided into the following three types: protopanaxadiol (PPD), protopanaxatriol (PPT), and oleanane. Typical PPD, also called 3β,12β,20-trihydroxydammar-24-ene type saponins, includes the ginsenosides Rb1, Rb2, Rc, and Rd. As shown in Fig. 2, PPD-type ginsenosides involve the attachment of the saccharide(s) C-3 and/or C-20. Rh2 and Rg3 are also PPD-type ginsenosides. In *P. ginseng*, the two most abundant PPT, also called 3β,6α,12β,20-tetrahydroxydammar-24-ene type saponins, are Re and Rg1. A variety of saponins can be biosynthesized via the O-glycosylation of PPT, which involves the attachment of saccharide(s) to C-6 and/or C-20. Typically, the hydroxyl group at C-3 remains free in PPT-type ginsenosides. Rf and Rh1 also belong to PPT-type ginsenosides.

The chemical name of ginsenoside compound K (C-K, Compound 1) is 20-O-β- D-glucopyranosyl-20(S)-protopanaxadiol; it is a non-natural protopanaxadiol ginsenoside. *P. ginseng* C. A. Mey, *P. quinquefolium* L, and *P. notoginseng* (Burk.) F. H. Chen, which contain the natural ginsenosides Rb1, Rb2, Rc, and Rd, are the main sources of C-K. C-K can only be produced through biotransformation; microbial fermentation and enzymatic methods are mainly used, with the latter being the preferred method. C-K is one of the major metabolites detected in blood after the oral administration of the ginsenosides Rb1, Rb2, or Rc; it is also speculated to be the major form of protopanaxadiol saponins absorbed through the intestine [10, 11]. Figure 2 shows the main components and structural formulae of ginsenosides that can affect bone remodelling [12–21].

## Bone remodelling

The bone is one of the most important tissues in the human body. Bone remodelling plays a critical role in maintaining the skeletal system and involves the processes of bone formation and bone absorption [22, 23].

There are two types of bone development. The first is intra-membrane osteogenesis, which includes the proliferation and differentiation of mesenchymal stem cells into pre-osteoblasts that differentiate into osteoblasts and secrete extracellular matrix. The cells are embedded into the calcification matrix turn into osteocytes, become ossification centers, and form bone trabeculae. The other type of bone development is intrachondral osteogenesis, which involves the proliferation and differentiation of mesenchymal stem cells into chondrocytes. The chondrocytes produce a cartilage matrix, which forms the cartilage and is gradually replaced with bone tissue. Osteoclasts are derived from hematopoietic stem cells and can perform bone resorption. Osteoblasts and osteoclasts complement each other and participate in bone development and remodelling. The entire process requires many intracellular signals as well as interactive molecules and signalling pathways to promote proliferation and differentiation.

### Osteogenesis and related molecules

**Fig. 3:**
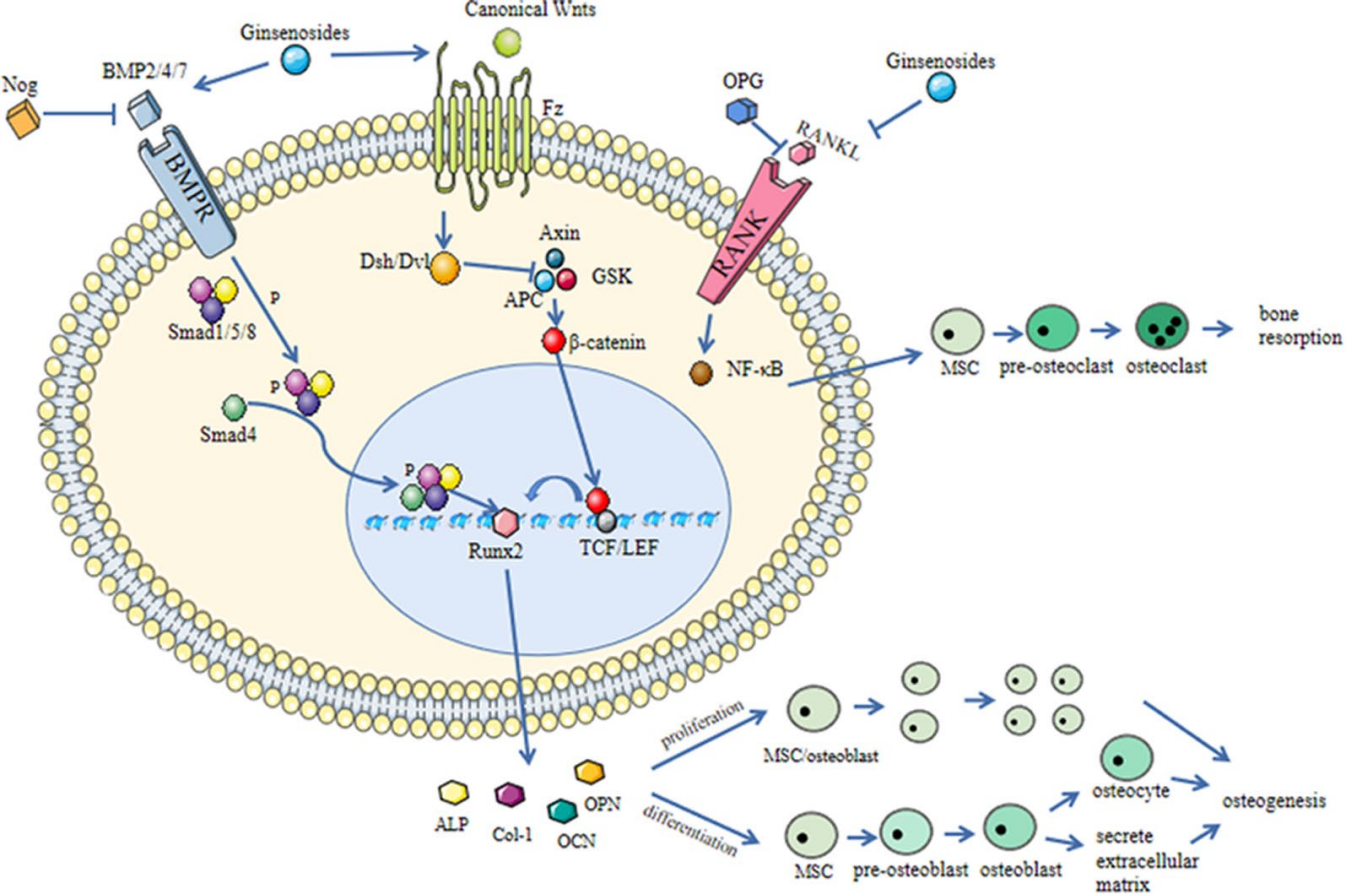
A brief schematic of the bone remodelling mechanism and the role of ginsenosides

The two most active pathways that regulate osteogenesis involve bone morphogenetic protein (BMP) and wingless/int-1 (WNT), as shown in Fig. 3.

The role of the Bone morphogenetic proteins (BMP) family, including BMP-2, BMP-4, and BMP-7, have been extensively studied in osteogenesis. There are two types of BMP receptors: BMPRI and BMPRII. Type I is a high-affinity receptor, while type II receptors bind specialized BMP ligands, including the three aforementioned BMPs [24]; both types are expressed on cell membranes. When BMP-2/4/7 binds to type II receptors, they activate type I receptors that in turn phosphorylate Smad1, 5, and 8 [25, 26], followed by the binding of the type I receptors to Smad4, the entry in the nucleus and recruitment of different transcription factors to regulate transcription. The Smad cascade can activate core-binding factor a1 (Cbfal), also known as Runt-related gene 2 (Runx2), which binds to the osteoblast-specific cis-acting element-2 (OSE2) of the promoter region of bone-specific genes and induces the expression of many bone marker genes [27], including alkaline phosphatase (ALP), osteocalcin (OCN), osteopontin (OPN), bone sialoprotein and bone-specific collagens. The expression of Runx2 marks the beginning of osteogenic differentiation; it is the first and most specific marker of bone formation [28]. ALP is an early phenotypic marker and an essential enzyme in osteoblast differentiation [29]. OCN is an important non-collagen component secreted by osteoblasts into the bone matrix during the mineral formation stage [30]; it is closely related to hydroxyapatite in bone tissue and participates in the regulation of bone calcium deposition. Col-1, one of the main components of the extracellular matrix secreted by osteoblasts, provides a structural framework for the maturation of the extracellular matrix and formation of calcified nodules [31–33]. In addition, BMP-2 can also induce ALP without Smad proteins by various pathways, such as the p38-mitogen-activated protein kinase (MAPK) [34]. Noggin (Nog) prevents BMP from binding to BMPR, thereby inhibiting the rate of bone formation.

WNT signalling consists of two major pathways: a canonical signalling pathway and a non-canonical signalling pathway. In canonical signalling, proteins such as Wnt3A, Wnt8 and Wnt10b bind to frizzled (Fz) receptors to generate dishevelled (DSH) and then transfer the signal to glycogen synthase kinase 3 (GSK-3), adenomatous polyposis coli (APC), axin and β-catenin. β-catenin enters the nucleus and binds to the lymphatic enhancer factor/T cell-specific transcription factor (TCF/LEF), so that the target gene is expressed [35, 36]. On the other hand, the non-canonical pathway involves intracellular secondary messengers: one to activate specific transcription factors through Ca^2+^ [37] and the other to control planar cell polarity (PCP) signalling. After binding to the Fz receptor, proteins activate Rho to regulate the cytoskeleton [38]. The canonical WNT pathway plays an important role in bone development. Wnt proteins are produced by osteoblasts and Wnt genes are upregulated during the osteogenic differentiation of BMSCs [39, 40]. Wnt10b, Wnt1, Wnt2 and Wnt3a regulate bone marker genes such as Runx2, ALP, Osx and OCN through canonical pathways [41]. Upregulation of Wnt signalling in osteoblasts can promote osteoblast proliferation and osteogenic activity [42]. The lack of Wnt10b or Wnt5a in mice leads to insufficient bone mass and abnormal bone tissue structure [43, 44]. In addition, non-canonical Wnt signalling also participates in osteogenesis and can interact with other signalling pathways [45]. For example, Wnt5a can promote osteogenic differentiation by promoting the Ror2-JNK and PLC-PKC-CaMKII pathways, while the former can stimulate RANKL in osteoclasts to regulate osteoclasis. This is of great significance for maintaining bone homeostasis [46–48]. The Wnt signalling pathway plays an important role in promoting the proliferation and differentiation of osteoblasts and stem cells. It can also promote bone formation by regulating the FGF and BMP pathways [49].

The BMP and WNT signalling pathways described above are active and play important roles in regulating osteogenesis.

### Osteoclast and related molecules

Osteoclasts are required for bone remodelling. RANK is a homotrimeric receptor that interacts with its ligand RANKL to induce downstream signal transduction, such as the activation of nuclear factor-kappa B (NF-κB), which can enter the nucleus to induce proliferation, differentiation and activation of osteoclasts [50]. The canonical Wnt pathway can produce OPG and inhibits the activity of osteoclasts by increasing the ratio of OPG to RANK [51]. Tartrate-resistant acid phosphatase (TRAP), mainly derived from osteoclasts, has a protective effect against tartaric acid. During the process of bone resorption, TRAP and other isoenzymes degrade the solid calcium phosphate mineralized substrate in the bone matrix. Moreover, TRAP marks the activity of osteoclasts and plays a role in osteoclastic bone resorption. Among the bone resorption indicators, TRAP has the highest specificity and is not easily affected by diurnal changes and liver or kidney diseases [52].

Therefore, changes in the content of the above molecules can objectively reflect the activity of osteoclasts and degree of activity of bone resorption.

### Relationship between bone development, fracture healing and osteoporosis

Bone development involves continuous bone remodelling which includes bone regeneration, enhancement, absorption and atrophy, to facilitate bone tissue growth and changes in its anatomical structure. The healing of fractures can be divided into three stages: acute inflammation, repair and remodelling. Inflammatory reactions include vasodilation, increased permeability, exudation of plasma proteins and inflammatory cell infiltration in the tissue of the fractured area during the acute inflammation phase. During repair, osteoblasts in the fractured area secrete collagen, synthesize the matrix and deposit calcium salts. In the bone remodelling phase, osteoblasts and osteoclasts work together to rebuild the bone trabecula of spongy bone along the direction of stress lines, strengthen the cortical bone, and connect the bone marrow cavity to restore the normal structure of the bone. The similarity between fracture healing and bone development is that they both undergo bone remodelling. The difference is that the former is a physiological process and the latter is a pathological process after trauma.

The balance between osteoclasts and osteoblasts results in bone homeostasis. However, in case of abnormal metabolism, bone metabolic diseases such as osteoporosis tend to occur. During osteoporosis, bone mass and bone density are reduced, and the microstructure of bone tissue is damaged, which results in increased bone fragility and risk of fracture. The treatment of osteoporosis also requires bone remodelling to promote bone formation and inhibit bone resorption.

## Effect of ginsenosides on bone remodelling

### Role and mechanism of ginsenosides in promoting osteoblast-related cell proliferation

In general, the ginsenosides Rb1, Rb2, Rg1, Rh1, Rg5, and Rk1 promote the proliferation of osteoblasts and stem cells [53–60]. Mesenchymal stem cells are multipotent cells capable of differentiating into osteoblasts and play an important role in the growth, development, and reconstruction of bone tissue. The research methods in this part mainly include MTT assay, CCK8, and [^3^H]-thymidine incorporation assay. Table 1 shows the role and mechanisms of ginsenosides in promoting osteoblast-related cell proliferation. The mixture of protopanaxadiol ginsenoside Rg5:Rk1 was obtained by Siddiqi et al. [60] by repeatedly cooking and drying fresh ginseng root. At present, there are few studies on the mechanism of ginsenosides promoting osteoblasts or MSCs. Ginsenoside Rg1 significantly increased the proportion of hDPCs and hDPSCs in the proliferative phase (S phase) and decreased the cells in the resting phase (G0/G1 phase) [57, 58]. Moreover, a gene expression profile microarray analysis was performed; results showed that compared to the control, ginsenoside Rg1 had more than 2000 gene expression differences. Through analysis, it was revealed that these genes are mainly related to the following functions: cell cycle, cell proliferation, growth factor and receptor activity, cellular metabolism, biosynthetic process, signal transduction, and apoptosis. The molecular mechanism of ginsenosides in promoting cell proliferation needs further study.

**Table 1:** Role and mechanism of ginsenosides in promoting osteoblast-related cell proliferation

Saponin	Cells	Research methods	Effect on cells	Dose-dependence	Mechanism	Refs.
1. Ginsenoside Rb1	Human adipose stem cells (hADSCs)	MTT assay	0.5 μmol/L Rb1 can significantly promote the proliferation of hADSCs, 6.0 μmol/L Rb1 has a significant inhibitory effect on proliferation	No		[53]
2. Ginsenoside Rb2	MC3T3-E1 cells	MTT assay	0.1, 1, 10 μmol/L Rb2 is non-toxic to cells and can inhibit cytotoxicity caused by H_2_O_2_ at 1 mM.	No		[54]
3. Ginsenoside Rg1	BMSC	CCK8	0.1, 1, 10 μg/mL Rg1 induced cell proliferation, the highest stimulation was in the 1 μg/mL group.	No		[55]
	Human periodontal ligament stem cells (hPDLSCs)	MTT assay	100 nmol/L to 10 μmol/L Rg1 can promote cell proliferation, and 100 μmol/L is cytotoxic.	Yes (100 nmol/L–10 μmol/L)		[56]
	Human dental pulp cells (hDPCs)	MTT assay, flow cytometry analysis	Rg1 can promote cell proliferation, especially in the 5 μmol/L group.	Yes (0.5, 2.5, 5 and 10 μmol/L)	Cells in S phase increased and cells in G0/G1 phase decreased	[57]
	Human dental pulp stem cells (hDPSCs)	[^3^H]-thymidine incorporation assay, flow cytometry analysis and gene expression profile microarray analysis	Rg1 can promote cell proliferation, especially in the 5 μmol/L group	Yes (0.5, 2.5, 5 and 10 μmol/L)	Cells in S phase increased and cells in G0/G1 phase decreased	[58]
4. Ginsenoside Rh1	MC3T3-E1 cells	MTT assay	Rh1 can promote cell proliferation	Yes (1–300 μmol/L)		[59]
5. Ginsenoside Rg5:Rk1	MC3T3-E1 cells	MTT assay	Rg5:Rk1 can promote cell proliferation	Yes (1–50 μg/mL)		[60]

### Effect and mechanism of ginsenoside on bone formation

Today, people are becoming increasingly interested in how drugs promote osteogenesis. The conventional in vitro research methods include ALP activity determination, calcium nodule staining, PCR and western blot, to detect the expression of osteogenesis-related factors. There are further studies on research methods. In vivo research methods include the establishment of animal models, imaging detection of bone reconstruction indicators, and the histological observation of bone structure. Many in vivo and in vitro experiments have shown that ginsenosides can up-regulate the expression of intracellular osteogenic transcription factors and osteogenic related gene products, induce osteogenic differentiation of pre-osteoblasts, stimulate osteoblast proliferation and promote bone nodule formation and matrix mineralization. They modulate various intracellular signalling pathways to exert these effects. Table 2 will introduce the roles and mechanisms of various major types of ginsenosides in bone remodelling.

**Table 2:** Roles and mechanisms of various major types of ginsenosides

Saponin	Osteogenesis				Osteoclastogenesis
	In vitro		In vivo		Cells, effect and mechanism
	Cells	Effect and mechanism	Animal model	Effect and mechanism	
1. Ginsenoside Rb1	hAPDSCs (ALP activity, calcium mineralization↑)	Dose-dependently promote the osteogenic activity of hADSCs (0.5–6.0 μmol/L) [53]			RAW264.7 cells (RANKL↓, TRAP staining and activity ↓); inhibit osteoclast differentiation [61]
	rMSCs(ALP activity↑, Runx-2, OCN, OPN, ALP expression↑)	Dose-dependently promote the osteogenic activity of rMSCs (0.01–1 μmol/L) [62]	OVX rats (serum analysis, mechanical testing, Masson Goldner trichrome staining, bone histomorphometry analysis)	No obvious effect on OVX rats [62]	Raw264.7 cells (c-Fos and NFATc1↓); inhibit osteoclast differentiation through JNK, p38 MAPKs and the NF-κB pathways [74]
2. Ginsenoside Rb2	MC3T3-E1 (ALP activity↑, calcium mineralization, mRNA expressions of ALP, Col-1, OCN and OPN↑)	Promote the differentiation of osteoblasts and resist oxidative damage caused by H_2_O_2_ [54]	OVX rats (ROS measurement, Van Gieson staining)	BMD↑, MDA↓, GSH↑, Rb2 may partially improve the microstructure and bone mass of trabecular OVX mice, prevent and treat osteoporosis [54]	MC3T3-E1(RANKL↓, IL-6↓) [48]; RAW264.7 cell, inhibit osteoclast differentiation through NF-κB-STAT3 signalling pathways↓ [75]
3. Ginsenoside Rc					RAW264.7 cells (RANKL↓, TRAP staining and activity ↓ ) [61]
4. Ginsenoside Rd	MC3T3-E1 (ALP activity, calcium mineralization↑, BMP-2 secretion↑, ALP, OCN, Col-1, BMP-2 expression↑)	Promotes osteogenesis through the AMPK-BMP-2 -smad signalling pathway [63]			BMMs (TRAP activity↓) inhibit osteoclast differentiation but cytotoxic [76]
5. Ginsenoside Re	MC3T3-E1 (ALP staining and activity, Runx-2, Col-1, OCN, OPN expression↑)	Promote osteoblast differentiation [64]	Zebrafish scales (Alizarin red S staining)	Promote mineralization of zebrafish scales [64]	RAW264.7 cells (RANKL↓, TRAP staining and activity ↓) [61] BMMs and zebrafish scales (TRAP staining and activity↓) inhibit osteoclast differentiation through ERK and RANKL-induced signalling pathway [76]
6. Ginsenoside Rf					RAW264.7 cells (RANKL↓, TRAP staining and activity ↓) [61]
7. Ginsenoside Rg1	BMSCs (ALP staining↑, calcification↑ BMP-2, Runx2, OCN, Col-1 and ALP expression↑)	Promote the osteogenesis through GR-dependent BMP/Smad signalling pathway [55]	Rat tibial fracture model (Micro-CT scanner HE, Safranin-O/Fast Green and immunohistochemical staining)	Promoted the transformation from the fibrous callus to osteogenic callus, increased bone strength and accelerated fracture healing [55]	RAW264.7 cells (RANKL↓, TRAP staining and activity ↓) [61]
	hPDLSCs (ALP activity↑, Runx-2, Col-1, OCN, OPN expression ↑)	Enhance osteogenic differentiation [56]			
	hDPCs (ALP activity↑ mineralized calcium nodules↑)	Enhance osteogenic differentiation [57]			
	hDPSCs (BMP-2, FGF-2 secretion↑, DSPP, ALP, OCN, BPM-2 and FGF2 mRNA↑)	The Roche Nimblegen Whole Human Genome Expression profile microarray; seven statistically significant pathways, gene expressions of DSPP↑ and DMP1↑ [58]			
8. Ginsenoside Rh1	MC3T3-E1 (ALP activity↑, Runx-2, Col-1, OCN expression↑, mineralized calcium nodules↑, glutathione contents↑)	Promote osteogenic differentiation and inhibit AMA-enhanced ROS [59]			
9. Ginsenoside Rh2(S)	MC3T3-E1 (ALP staining, calcification↑ ALP, Runx2, OSX, OCN, OPN and Col-1 expression↑)	Stimulated the differentiation and the mineralization through PKD/p38 MAPK and PKD/AMPK signalling pathway [70, 71]			
10. C-K	MC3T3-E1 (ALP activity, Col-1 content, and mineralization ↑ ALP, Runx2 and Col-1 expression↑)	Inhibited H_2_O_2_-induced ROS NO production and inflammation; stimulated osteoblast differentiation [72]			
	MC3T3-E1 (ALP activity, ALP, Col-1, and Runx2 expression↑)	Induce osteogenic differentiation through WNT signalling pathway [73]			
11. Rg5:Rk1	MC3T3-E1ALP activity↑, calcification↑ BMP-2, Runx2 and Col-1 expression↑	They speculated that osteogenesis-promoting effect is achieved through the BMP-2/Runx2 pathway [60]			
12. Ginseng water extract: 1.19% Rb1, 0.12% Rb2, 0.57% Rg1, 0.07% Rc, 0.64% Re, and 0.04% Rf			OVX (µ-CT, Bone histomorphometric analysis)	Ginseng can prevent bone loss and trabecular microstructure deterioration caused by OVX. Ginseng may be a good drug for the prevention and treatment of postmenopausal osteoporosis [61]	RAW264.7 cells (RANKL↓, TRAP staining and activity ↓) The ginseng water extract and the five ginsenosides except Rb2 can inhibit osteoclast differentiation [61]

↑: up-regulation ↓: down-regulation

#### Ginsenoside mixture

At present, there are only a few studies on the osteogenesis of ginsenoside mixtures. Siddiqi et al. obtained ginsenoside mixture Rg5:Rk1 after repeatedly cooking and drying fresh ginseng root [60]. Ginsenosides Rg5:Rk1 can promote osteogenic differentiation of MC3T3-E1 cells by increasing the activity of ALP, the content of Col-1 and the intracellular calcium deposition. Siddiqi et al. speculated that this osteogenesis-promoting effect is achieved through the BMP-2/Runx2 pathway [60]. Lee et al. [61] used chromatography to verify that the main part of ginseng water extract included 1.19% Rb1, 0.12% Rb2, 0.57% Rg1, 0.07% Rc, 0.64% Re and 0.04% Rf. It was found that in ovariectomized (OVX) rats treated with ginseng water extract, the sharp decrease in bone mineral density (BMD) and the deterioration of trabecular bone structures can be significantly reduced. In the presence of ginseng water extract, other bone remodelling markers such as Tb.N, Tb.Th and Tb.Sp were also significantly restored. Hence, ginseng can prevent bone loss and trabecular microstructure deterioration caused by OVX and is a potential drug for the prevention and treatment of postmenopausal osteoporosis. Lee and Siddiqi have proved the role of ginseng mixture in promoting bone tissue. However, the question remains whether the individual ingredients exert similar effects.

#### Ginsenoside Rb1

Ginsenoside Rb1 can promote the osteogenic activity of hADSCs and rMSCs in a dose-dependent manner (0.5–6.0 μmol/L and 0.01–1 μmol/L), and promote ALP activity, mineralization, and expression of osteoblast-related proteins [53, 62]; it however, has no significant effect on bone loss in OVX rats. However, in their experiments, the high-dose Rb1 group was injected with 6 mg/kg/day, and an increase in the dose could be considered in animal experiments in the future.

#### Ginsenoside Rb2

Ginsenoside Rb2 (0.1–10 μmol/L) can improve the ALP activity; it increases the degree of calcium mineralization and mRNA expression of ALP, Col-1, OCN, and OPN to resist oxidative damage caused by H_2_O_2_ in MC3T3-E1 cells [54]. Besides, Rb2 reduced the expression levels of receptor activator of nuclear factor kappa-B ligand (RANKL) and IL-6 and inhibited the H_2_O_2_-induced production of ROS. In vivo studies showed that in OVX mice, the continuous administration of Rb2 for 12 weeks partially decreased the malondialdehyde (MDA) activity in the blood and increased the activity of reduced glutathione (GSH). MDA is a parameter for assessing the state of oxidative damage, and GSH is an intracellular oxidant, which can relieve the oxidative stress of cells. In addition, Rb2 improved the microstructure of the trabecular bone and increased BMD of the fourth lumbar spine (L4) and the distal femur.

#### Ginsenoside Rd

Ginsenoside Rd can improve the ALP activity and mineralization ability of MC3T3-E1 cells. The expression levels of ALP, OCN, Col-1, and BMP-2 can be increased, and the mRNA expression level of BMP-2 can be inhibited by noggin, AMPK inhibitor (Ara-A) or transfection of smad4-targeted siRNA. Therefore, Kim believes that ginsenoside Rd promotes osteogenesis through the AMPK-BMP-2-Smad pathway [63].

#### Ginsenoside Re

Ginsenoside Re can promote osteoblast differentiation and mineralization of zebrafish scales. Ginsenoside Re was non-cytotoxic to MC3T3-E1 cells and enhanced ALP staining and activity, as well as mRNA levels of osteoblast markers Col-1, Alp and OCN in MC3T3-E1 cells. The calcium concentration of ginsenoside Re-treated zebrafish scales was detected by Alizarin Red S staining [64].

#### Ginsenoside Rg1

At present, there are more and more in-depth studies on ginsenoside Rg1. It is known to promote the osteogenic differentiation of hPDLSCs [56], hDPCs [57], rBMSCs [55], as well as the ALP activity and the formation of mineralized calcium nodules. It can not only stimulate the secretion of BMP-2 and FGF-2 from hDPSCs [58], but also promote the protein expression of DSPP, ALP, BMP -2, FGF-2, Runx-2, Col-1, OCN and OPN [55–58]. Wang et al. [58] further compared the representative gene expression profiles of DPSCs using Roche’s full human genome expression profile microarray chip. In the ginsenoside Rg1 (5 μmol/L) group, there were 1498 upregulated genes and 561 downregulated genes. Pathway analysis found seven statistically significant pathways, such as the cell cycle pathway, the MAPK signalling pathway and the TGF-β signalling pathway. Moreover, Gu et al. [55] further explored the mechanisms and found that Rg1 promotes the osteogenesis of rBMSCs through the GR-dependent BMP/Smad-signalling pathway. In addition to the progress of in vitro experiments, in vivo experiments were performed. A rat tibial fracture model was established, and a Micro-CT scanner revealed that Rg1 stimulates fracture healing. Then H&E staining and Safranin O/Light green red staining were used to examine the bone section. It was found that Rg1 promoted the transformation from fibrous to osteogenic callus, increased bone strength and accelerated fracture healing. These results suggest that Rg1 promotes cartilage calcification and osteogenesis at a later stage. Whether or not Rg1 can regulate bone metabolism through other signal pathways that Wang analyzed needs further verification.

#### Ginsenoside Rh1

Ginsenoside Rh1 can promote osteogenic differentiation and stimulate ALP activity of MC3T3-E1 cells [59], promote mineralization and increase glutathione content. Rh1 was also found to increase BMP-2, Runx2, ALP, Col-1 and OCN expression levels. During mitochondrial electron transport, reactive oxygen species (ROS) keep cells in a state of oxidative stress by producing high levels of oxidants, which destroy proteins, lipids and DNA [65]. Oxidative stress may also destroy osteoblasts and cause osteoporosis [66, 67]. New evidence suggests that ROS increases bone resorption by enhancing osteoclast development and activity [68]. ROS also cause apoptosis and reduce their activity, which leads to osteoblasts apoptosis [69]. Additionally, Ginsenoside Rh1 shows an inhibitory effect on AMA-enhanced ROS production levels in MC3T3-E1 cells. The level of glutathione after Rh1 treatment significantly increases in a concentration-dependent manner, indicating that the increase in the activity of osteoblasts induced by Rh1 was related to the increase in glutathione content. AMA-treated MC3T3-E1 cells significantly increased ROS production, while Rh1 treatment strongly inhibited this effect of AMA.

#### Ginsenoside Rh2 (S)

Ginsenoside Rh2 (S) stimulated the differentiation and the mineralization of osteoblasts MC3T3-E1, which were expressed by their differentiation markers (ALP, Runx2, OSX, OCN, OPN and Col-1) and upregulation of von Kossa/Alizarin red staining [70, 71]. Ginsenoside Rh2 (S) activated protein kinase D (PKD), p38 mitogen-activated protein kinase (MAPK) and AMP-activated protein kinase (AMPK) in a time- and concentration-dependent manner, which can be inhibited by corresponding inhibitors. Therefore, Rh2 (S) induced differentiation and mineralization of MC3T3-E1 cells by activating the PKD/p38 MAPK signalling pathway [70] and the PKD/AMPK signalling pathway [71].

#### C-K

C-K inhibited H_2_O_2_-induced ROS and NO production in MC3T3-E1 cells in a dose-dependent manner. Cultured H_2_O_2_ stimulated MC3T3-E1 cells exposed to C-K showed a sharp increase in the expression of osteoblast differentiation markers, such as ALP activity, Col-1 content and mineralization. In addition, C-K reduced inflammation-related genes including IKK and interleukin 1β (IL-1β) expression [72]. Zhou et al. [73] converted four compounds from ginsenoside Rb1, including C-K, compounds 2, 3, and 4. The compounds significantly increased ALP activity in a dose-dependent manner. The mRNA expression of the osteoblast differentiation markers ALP, Col-1, and Runx2 increased significantly in a dose-dependent manner. C-K and these new derivatives significantly upregulated the mRNA expression of genes of Wnt/β-catenin signalling pathway-related regulators, including Wnt10b, Wnt11, Lrp5 and β-catenin. These minor ginsenosides can induce osteogenic differentiation of MC3T3-E1 cells by partial or independent controlling of the classical Wnt signalling pathway.

The above section introduces the role of various types of ginsenosides in promoting bone formation, such as up-regulating the expression of intracellular osteogenic gene products, inducing osteogenic differentiation, promoting bone nodule formation and matrix mineralization, and regulating various intracellular signal pathways.

### Ginsenosides inhibit the mechanism of osteoclastogenesis

Ginseng water extract and 14 kinds of ginsenosides (Rb1, Rb2, Rb3, Rg1, Rg2, Rg3, Rc, Rd, Re, Rf, CK, F11, Rh1, Rh2) can slow bone resorption. During bone resorption, ginsenosides inhibit osteoclast differentiation, TRAP activity and staining, the activation of signalling pathways such as NF-κB induced by RANKL. Among them, Rb1, Rb2 and Re have better effects, and their mechanism of action has been further explored.

#### Ginsenoside mixture

Ginseng extract effectively inhibits RANKL-induced osteoclast differentiation. Lee et al. [61] exposed RAW 264.7 cells to RANKL and M-CSF receptor activator for 5 days, and each group was added with different concentrations of ginseng water extract. It was found that Rb1, Rg1, Rc, Re, and Rf can inhibit osteoclast differentiation, TRAP activity and staining.

#### Ginsenoside Rb1

Similar results were found for the independent components of ginsenosides. Ginsenoside Rb1 can inhibit osteoclastogenesis by inhibiting RANKL-induced activation of JNK, p38 MAPKs and the NF-κB pathways, thereby downregulating the gene expression of c-Fos and NFATc1 in osteoclast precursors [74].

#### Ginsenoside Rb2

The inhibitory effect of Rb2 on osteoclast differentiation may be related to blocking the NF-κB and STAT3 signalling pathways [75]. Rb2 can promote OPN expression and bone resorption. On the other hand, Rb2 inhibits the formation of TRAP-positive multinucleated cells and the expression of TRAP in a dose-dependent manner, and significantly inhibits RANKL-induced NF-κB activation. Furthermore, Rb2 can also significantly inhibit the expression of osteoclast marker genes, including NFATc1, c-Fos, OSCAR and cathepsin K nuclear factors. In addition, knocking down STAT3 can significantly enhance the inhibitory effect of Rb2 on osteoclast differentiation, which indicates that Rb2 also remarkably inhibits the activation of signal transductors and the STAT3 signalling pathway.

#### Ginsenoside Re

Park et al. [76] treated RANKL-induced bone marrow-derived macrophages (BMMs) with 14 kinds of ginsenosides (including the 14 above) and osteoclast differentiation was evaluated by TRAP activity. To some extent, TRAP activity in all the 14 ginsenoside groups was inhibited. At a concentration of 2.5 μmol/L, ginsenosides Rd, Re, C-K and fraction 11 inhibited osteoclast differentiation by about 60%. All types of ginsenosides except ginsenoside Rd were found to be non-toxic. Among various ginsenosides, ginsenoside Re showed the strongest inhibitory effect on osteoclast differentiation. The mechanism of Re was further explored and it was found that ginsenoside Re affects osteoclast differentiation by inhibiting the RANKL-induced signalling pathways. In particular, ginsenoside Re blocked ERK signalling, a known mechanism of osteoclast differentiation. Re was applied to a zebrafish model to study its effects on osteoclast differentiation in vivo. The results demonstrated that ginsenoside Re inhibited osteoclast differentiation, and osteoclast marker genes were significantly reduced in zebrafish scales. Treatment with Re reduced the mRNA expression levels of TRAP and cathepsin K, although it did not significantly affect the expression of osteoblast marker genes.

In summary, ginsenosides can inhibit osteoclast differentiation and slow bone resorption. The effects of Rb1, Rb2 and Re have been well described; the effect and mechanism of other types of ginsenosides on bone resorption need to be further explored.

### Other effects related to bone remodelling

As earlier discussed, there are two ways of bone formation—intra-membrane osteogenesis, and intra-chondral osteogenesis. Having discussed how ginsenosides promote intra-membrane osteogenesis, we turn our attention to the potential effects of ginsenosides on cartilage.

Ginsenosides F4 and Rg3 prevent cartilage destruction in rabbit cartilage tissue culture. Lee et al. [77] studied how 11 kinds of ginsenosides (Rb1, Rb2, Rc, Rd, Re, Rf, Rg1, Rg3, Rg5, Rk1 and F4) affect the induction of MMP-13 in the human chondrocyte cell line SW1353. In osteoarthritis, MMP-13 plays an important role in the degradation of major collagens embedded into the cartilage. Among them, ginsenosides Rc, Rd, Rf, Rg3 and F4, were found to inhibit the expression of MMP-13 in IL-1β-treated SW1353 cells at a non-cytotoxic concentration (1–50 μmol/L). The most prominent inhibitors are ginsenosides F4 and Rg3. Ginsenoside F4 was found to strongly inhibit p38 mitogen-activated protein kinase (p38 MAPK) activation in the signalling pathway. Ginsenosides F4 and Rg3 also reduced the release of glycosaminoglycan in rabbit joint cartilage culture treated with IL-1α. The mechanism of action of ginsenosides on cartilage remains to be fully elucidated.

Dental tissue and bone tissue are homologous in tissue origin (both derived from neural crest). Ginsenoside can promote bone remodelling in bone tissue, and some studies proposed similar effects in tooth tissue.

Ginsenoside Rg1 promotes the differentiation of hDPCs and increases the expression of DSPP and DMP1 genes [57]. DSPP is a type of extracellular matrix protein of dentin. It is considered as an odontoblastic marker, and can trigger the mineralization of dentin. DMP1, a non-collagen matrix protein, is an important component of mineralized tissues and can be expressed in the bone, dentin, and cementum. DMP1 plays an important role in regulating the differentiation of odontoblasts, formation of dentin tubules and mineralization of dentin. These two genes can promote the differentiation of dental pulp cells into odontoblasts and dentin regeneration. Therefore, ginsenoside Rg1 may become a new pulp capping agent, which will be important in dental pulp biotherapy and can provide new strategies for preventing and treating dental caries. Further detailed studies of ginsenoside Rg1 are needed.

## Conclusion and outlook

Bone homeostasis is tightly regulated to retain a balance between bone formation and bone resorption. Many anabolic drugs are used as bone-targeting therapeutic agents for the promotion of osteoblast-mediated bone formation or the inhibition of osteoclast-mediated bone resorption. The functions and mechanisms of various types of ginsenosides in bone remodelling are shown in Table 2 and Fig. 3. Many ginsenosides can promote bone formation and inhibit bone resorption, such as Rb1, Rb2 and Re. They play an important role in promoting bone remodelling. These ginsenosides have multiple beneficial roles in bone remodelling. In addition to promoting bone formation and inhibiting bone resorption, they also have anti-inflammatory and antioxidant effects, which can alleviate oxidative stress. These functions are complementary to bone reconstruction.

Many future directions can be taken with this. Firstly, there is little research on the mechanism of ginsenosides on the proliferation of BMSC or osteoblasts, which can be further explored. Secondly, since ginseng from different sources and processing methods have different extracted components, a novel approach for maintaining stability needs to be found. Thirdly, although it is known that Rb1 has the effect of promoting osteogenesis in vitro, whether or not it promotes the formation of bone in animals is still undiscovered. At present, the effect of Rb1 on OVX rats is relatively small, perhaps because the dose is insufficient, and increasing the dose could be a consideration in future research. Fourthly, the role of Rc in bone reconstruction has not been studied in detail and it may be worth further exploration. Many pathways are involved in Wang’s research [58], and further verification is needed. Further, there are many in vitro studies on various types of ginsenosides affecting bone remodelling, but in vivo studies need to be conducted to corroborate the in vitro findings. Lastly, the current research on ginsenosides in the oral cavity has only explored the promoting effect of Rg1 on dentin. In the future, whether other types of ginsenosides have similar effects and more functions is worthy of in-depth study.

With the growing interest in bone tissue engineering, the selection of appropriate cytokines for bone remodelling has become a hotspot of research. In recent years, the effect of ginsenosides on bone remodelling has received increasing attention. Ginsenosides have the potential to be new drugs for the treatment of osteoporosis, promote fracture healing and are strong candidates for cytokines in the tissue-engineered bone.

## Data Availability

Not applicable.

## Abbreviations

ALP: Alkaline phosphatase
Runx2: Runt-related gene 2
OCN: Osteocalcin
OPN: Osteopontin
Col-1: Type I collagen
TRAP: Tartrate-resistant acid phosphatase
ROS: Reactive oxygen species
MSCs: Mesenchymal stem cells
hADSCs: Human adipose stem cells
hPDLSCs: Human periodontal ligament stem cells
hDPCs: Human dental pulp cells
hDPSCs: Human dental pulp stem cells
BMD: Bone mineral density
OVX: Ovariectomized
MDA: Malondialdehyde
GSH: Glutathione
